# A potentially lethal interaction: Migraine, human immunodeficiency virus and ergotism – A primary care case report

**DOI:** 10.51866/cr.748

**Published:** 2025-02-08

**Authors:** Ismail Ahmad Sahli Mahzuz, Ismail Shaiful Bahari, Lili Husniati Yaacob

**Affiliations:** 1 MD, MMed (Family Medicine), Senior Consultant of Family Medicine, Department of Family Medicine, School of Medical Sciences, Universiti Sains Malaysia, Kubang Kerian, Kelantan, Malaysia. Email: shaifulb@usm.my, shaifulbahari67@gmail.com; 2 MBBS, Department of Family Medicine, School of Medical Sciences, Universiti, Sains Malaysia, Kubang Kerian, Kelantan, Malaysia.; 3 MBBS, MMed (Family Medicine), Department of Family Medicine, School of Medical Sciences, Universiti, Sains Malaysia, Kubang Kerian, Kelantan, Malaysia.

**Keywords:** Ergotism, Migraine, Drug interaction, HIV, Cytochrome P450

## Abstract

Ergotism is a rare but potentially serious condition characterised by peripheral vasospasm. Its diagnosis is challenging because the presentation varies depending on the type and location of the affected blood vessels. Ergot alkaloids, including ergotamine, are metabolised by the cytochrome P450 isoenzyme CYP3A4. Concurrent use of ergotamine with CYP3A4 inhibitors can significantly increase the risk of ergotism. However, this potentially dangerous drug interaction is often underestimated in general practice. Herein, we report the case of a middle- aged woman with a history of migraine headaches, who was treated with Cafergot (ergotamine tartrate and caffeine). After the initiation of human immunodeficiency virus therapy with Kaletra (lopinavir/ritonavir), she experienced recurrent episodes of bluish discoloration, livedo reticularis and tingling sensation in her upper and lower extremities over several years. Despite multiple hospital visits and extensive diagnostic workups, including normal blood investigations and biopsy, the correct diagnosis of ergotism-induced vasospasm due to ritonavir—ergotamine interaction was delayed. This diagnosis was supported by CT angiography, which demonstrated vasospasm of the femoral arteries. The patient’s symptoms significantly resolved following ergotamine discontinuation. Ergotism is a self-limiting condition that can be fatal if not recognised and treated promptly. This case highlights the importance of awareness, particularly in primary care settings, on the potential drug interaction, principally in patients receiving drugs that inhibit CYP3A4, such as protease inhibitors. Clinicians should have a low threshold for suspecting ergotism in patients with recurrent or unexplained limb pain, numbness and skin changes, especially if they have a history of ergot alkaloid use.

## Introduction

Ergotamine is commonly used in outpatient settings as a treatment for migraine headaches. Ergotamine-induced vasospasm is a rare condition and can be influenced by interactions with other drugs that may potentiate their effect. Clinically, ergotism can cause arterial vasospasm, particularly in the lower extremities.^1^ Ritonavir, a component of protease inhibitors, is widely used in antiretroviral therapy for human immunodeficiency virus (HIV) infection. It is known to inhibit cytochrome P450 (CYP3A4).^2^ As a potent CYP3A4 inhibitor, ritonavir can significantly elevate plasma concentrations of ergotamine, which is metabolised by this enzyme. This drug—drug interaction increases the risk of ergotism and can potentially cause life- threatening vasospasm when these medications are administered together. This case report highlights a significant condition of ritonavir— ergotamine interaction-induced vasospasm, emphasising the importance of drug interaction awareness in clinical practice. This report aims to meaningfully contribute to the existing body of literature and enhance patient safety by discussing the diagnosis, clinical presentation and implications of this interaction.

## Case presentation

A 30-year-old woman presented to the emergency department with a 1-week history of bilateral foot numbness, associated with bluish discoloration and a painful tingling sensation for 2 days. The condition was aggravated by cold weather. Otherwise, she denied constitutional symptoms, connective tissue disease manifestations or thromboembolic history.

Prior to the current presentation, the patient had recently taken two tablets of ergotamine. She was a non-smoker with a long history of migraines with aura, occurring frequently, about once a month, characterised by a left-sided headache, aura, photophobia, vomiting and blurry vision, lasting less than 24 hours. These symptoms were treated with ergotamine tablets, which had been prescribed by her general practitioner for over 5 years.

**Figure 1.**
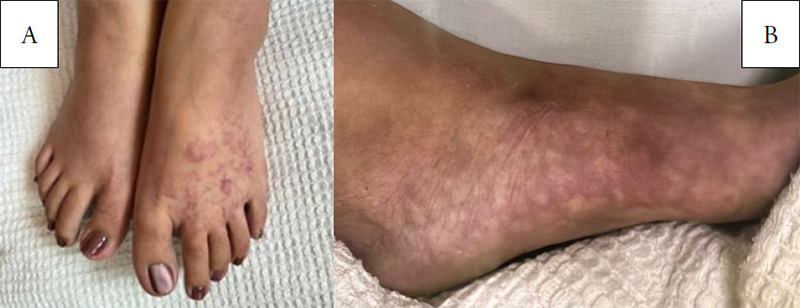
(A) The anterior aspect of the bilateral foot shows skin mottling (livedo reticularis). (B) The medial aspect of the left foot shows prominent skin mottling (livedo reticularis).

The patient also had an underlying HIV infection, diagnosed 3 years ago, and was receiving highly active antiretroviral therapy (HAART). She was initially started on Combivir (lamivudine/zidovudine), but the medication was changed to lopinavir/ritonavir 2 years ago due to anaemia. Since starting on lopinavir/ritonavir (200/50 mg) BID, she had experienced recurrent episodes of numbness in both hands and feet associated with skin discoloration, characterised by skin pallor, cyanosis and pink discoloration. These episodes, which led to multiple hospital visits, were usually triggered by taking ergotamine. On presentation at the casualty, she was alert, had pinkish conjunctiva, was not tachypnoeic and had no facial asymmetry. Her blood pressure was 112/86 mmHg; pulse rate, 82 beats per minute; and respiratory rate, 20 breaths per minute. The oxygen saturation level was documented as 95% on room air. Both her hands and feet were cold to touch, with bluish discoloration and skin mottling over the bilateral feet, with more dominance on the left foot (Figure 1).

The sensation was reduced in both hands and feet. The pulses of the radial, ulnar, popliteal and tibial arteries were diminished bilaterally, with more significant reduction over the left side. Electrocardiogram and echocardiogram showed no abnormalities.

**Figure 2.**
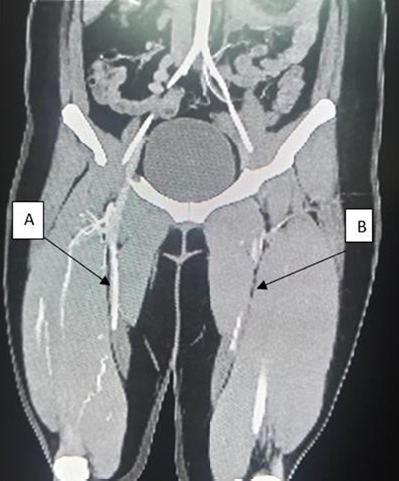
CT angiography of the bilateral lower limb (coronal view) shows significant narrowing from the middle to the distal segments of the bilateral superficial femoral arteries, worse on the left femoral artery. (A) Right femoral artery. (B) Left femoral artery.

Blood investigations showed normal full blood count, liver function and kidney function. Autoimmune markers including ANA, c-ANCA, p-ANCA, ds-DNA, anti-MPO, anti-PR3, anti-B2GP1, anticardiolipin and lupus anticoagulant were negative, ruling out systemic autoimmune disorders. The Coombs test, G6PD test and coagulation profile test yielded normal findings. A skin biopsy revealed no inflammation or vasculitis. In addition, her HIV RNA viral load was undetectable. These results suggested no evidence of autoimmune, haematological or infectious disease. An urgent CT angiography of the bilateral lower limb revealed progressive symmetrical narrowing of the bilateral arterial supply, worse on the left side, consistent with peripheral vascular spasm (Figure 2).

Ergotism-induced vasospasm was diagnosed after reviewing all blood investigations, histopathological findings and radiological reports. The patient was admitted to the ward, where ergotamine was discontinued and replaced with flunarizine and naproxen 550 mg PRN during migraine attacks. Her HAART was continued at the same dosage. During her stay in the ward, the patient’s symptoms significantly improved, and her pulses were gradually restored. She was discharged from the hospital and seen on day 10 post-discharge at the outpatient clinic with no complaints or abnormalities found.

## Discussion

Ergot is derived from the *Claviceps purpurea fungus.* Ergotism can occur when this fungus is ingested through contaminated grain, such as rye, or through the use of medications containing ergot derivatives, such as ergotamine. Ergotamine has been used for the treatment and prevention of migraine headaches for decades.^3,4^ Ergotamine-induced vasospasm due to drug interactions is rare, with an estimated incidence of 0.01%-0.02%.^5^ Ergot toxicity is most commonly seen in women with migraine headaches in their mid-30s.^6^

Ergotamine is commonly administered in combination with caffeine due to its low oral bioavailability. Once absorbed, ergotamine is metabolised by cytochrome P450 enzymes (CYP3A4).^7^ The toxicity of ergotamine can be potentiated by interactions with other drugs that inhibit cytochrome P450 enzymes, particularly CYP3A4, such as ritonavir, which is a component of protease inhibitors. At the vascular level, this ergot alkaloid acts on serotonin receptors (5-HT receptors) and alpha- adrenergic receptors in vascular smooth muscles, inducing vasoconstriction and vasospasm.

Several reports have documented interactions between ergotamine and ritonavir, leading to severe peripheral vasoconstriction, particularly in the lower limbs. This is because of anatomical and physiological factors, such as the effects of gravity on blood flow and the greater distance of the lower limbs from the heart. In contrast, the upper limbs, being closer to the heart, are less susceptible to these effects.^8^ In a previous study, severe ergotism was noted in a patient taking ritonavir even after a single dose of ergotamine.^9^ Another study reported that in 23 cases of ergotism associated with HIV protease inhibitors, 22% of patients experienced serious complications.^10^

The presentation of ergotism varies depending on the sites of vessel involvement. The clinical manifestations of ergotism with vasospasm of the lower limbs include pallor, cyanosis, paraesthesia, coldness, livedo reticularis and calf pain. In severe cases, patients may present with gangrene and ulceration requiring amputation.^11^ If ischaemia involves multiple territories, such as the cerebral, coronary, carotid, mesenteric, renal and pelvic arteries, patients may experience headaches, abdominal pain and lumbar pain.^1^ In our case study, the patient exhibited classic signs of ergotism, including bluish discoloration of both limbs, paraesthesia and livedo reticularis. However, these symptoms can also occur in patients with vasculitis and autoimmune diseases. Skin biopsy plays a critical role in diagnosing vasculitis and is indicated when clinical features strongly suggest the presence of cutaneous vasculitis.^12^

Diagnosing ergotism-induced vasospasm requires a high degree of suspicion, particularly regarding patients’ medication history, especially in those with migraine headaches.^13^ In this case, the diagnosis was made after multiple hospital visits and a comprehensive evaluation. The diagnosis was mainly clinical and supported by the evidence of vasospasm observed on CT angiography. Differential diagnoses were ruled out based on several factors: a) The patient had no risk factors for atherosclerosis, such as comorbidities or a history of smoking; b) there was no evidence of vasculitis, as confirmed by both biochemical tests and histopathological examination; c) the laboratory findings were normal for autoimmune markers; and d) there was no history of occupational exposure, e) infection such as fever or sepsis and f) medication allergies, malignancy and paraneoplastic syndrome. We believe that ritonavir-ergotamine interaction was the cause of this symptom phenomenon.

The mainstay treatment of the condition is an immediate cessation of ergot products.^1^ Drug history is therefore a crucial component of patient history. Intravenous hydration, combined with low-molecular-weight heparin and a direct arterial vasodilator such as nitroprusside or nitrate, has been found to be beneficial in the acute treatment of ergotism.^1^ Recovery should occur within 4 days after cessation of ergot-containing medication.^1^

In our case, the patient recovered within 48 hours after ergotamine discontinuation, whereby the pulses gradually returned, and livedo reticularis improved. Clinicians should be aware that there are alternatives to ergotamine for the treatment of migraine attacks. Triptans are recognised as the gold standard option, and the use of NSAIDs has been proven effective in treating acute migraines.^14^

## Conclusion

The co-administration of ritonavir and ergotamine may increase the risk of developing ergotism. Healthcare providers, particularly in primary care settings, must recognise the potential effects of these groups of drugs that can result in devastating drug interactions, especially in individuals with migraines and underlying HIV infection. The definitive treatment is to discontinue ergot derivatives. Ergotism is a self-limiting condition, and the prognosis is good. This case emphasises the necessity for comprehensive medication reviews and patient education regarding the potential risks of drug interactions.
